# Supplementary material for: Trends of Stunting Prevalence and Its Associated Factors among Nigerian Children Aged 0–59 Months Residing in the Northern Nigeria, 2008–2018
Source: Nutrients. 2021 Nov 29;13(12):4312. doi: 10.3390/nu13124312 (PMC8708583; doi:10.3390/nu13124312)
Supplement: Supplementary file 1 [file nutrients-13-04312-s001.zip › SupplementaryTable S1-nutr.pdf]

Table S1: Potential associated factors and its stunting prevalence among children aged 0—59 months by survey year in the NGZs, Nigeria (2008—2018)

**Variable**	Weighted total (N)	2008 NDHS PR(95% CI)	Weighted total (N)	2013 NDHS PR(95% CI)	Weighted total (N)	2018 NDHS PR(95% CI)
**Community level factor**						
**Residence type**						
Urban	2533	43.2 (40.2—46.3)	3944	36.8 (33.8 —39.9)	1865	38.2 (34.7 —41.9)
Rural	8340	51.2 (49.6—52.8)	12209	48.9 (47.0— 50.8)	4793	51.6 (49.5 —53.6)
**Geopolitical zones (North)**						
North Central	2562	43.4 (40.6—46.2)	3562	28.9 (26.3 —31.6)	1597	28.1 (24.9— 31.6)
North East	2991	48.8 (46.1—51.4)	4086	42.2 (39.3 —45.1)	1745	48.6 (45.5— 51.8)
North West	5319	52.6 (50.4—54.7)	8505	54.9 (52.5 —57.2)	3316	56.9 (54.3 —59.4)
**Northcentral**						
Benue	649	35.8 (32.4— 39.4)	784	21.5 (16.7— 27.3)	365	20.0 (15.5— 25.5)
Kogi	293	36.0 (30.2 —42.2)	332	22.8 (18.0 —28.5)	145	20.5 (15.2— 27.0)
Kwara	308	50.6 (41.8 —59.4)	344	27.7 (22.6 —33.5)	173	31.6 (21.8 —43.3)
Nasarawa	229	42.9 (36.9— 49.2)	368	33.9 (28.1 —40.3)	179	31.3 (25.4 —37.9)
Niger	489	45.6 (40.3— 51.0)	1145	33.5 (28.4— 38.9)	462	28.3 (21.4 —36.4)
Plateau	394	59.8 (51.7— 67.4)	408	35.5 (29.3 —42.1)	195	45.2 (37.2 —53.4)
**Northeast states**						
Adamawa	500	42.8 (37.4 —48.3)	588	33.0 (28.2— 38.3)	241	37.3 (29.5— 45.9)
Bauchi	730	50.6 (45.0 —56.2)	1017	50.5 (46.0 —55.1)	414	56.1 (49.8— 62.3)
Borno	690	49.4 (42.0 —56.8)	716	26.8 (22.4— 31.7)	381	45.4 (39.2 —51.7)
Gombe	304	52.0 (47.9 —56.1)	463	47.2 (41.7— 52.7)	197	50.5 (42.9— 58.1)
Taraba	372	42.8 (37.6— 48.1)	625	42.9 (36.5— 49.7)	203	40.9 (32.3 —50.0)
Yobe	395	54.9 (49.7— 59.9)	678	49.7 (44.4 —55.0)	310	55.3 (47.2— 63.2)
**Northwest states**						
Jigawa	651	53.2 (48.5— 58.0)	1079	59.2 (55.1 —63.3)	383	63.6 (57.9— 68.9)

Zamfara	417	54.4 (48.5— 60.2)	1247	55.6 (52.0— 59.2)	357	50.7 (43.7 —57.7)
Katsina	868	59.2 (54.3 —64.0)	1280	59.0 (52.1— 65.6)	668	60.3 (53.5 —66.8)
Sokoto	704	53.0 (48.9 —57.1)	875	51.3 (47.3 —55.3)	249	53.6 (46.3— 60.9)
Kano	1497	45.7 (40.6 —50.8)	2214	48.4 (42.3 —54.5)	713	57.1 (51.9— 62.0)
Kaduna	908	52.6 (48.2 —56.9)	994	56.5 (50.0— 62.9)	642	49.2 (43.5— 55.0)
Kebbi	274	63.7 (57.4— 69.6)	815	60.7 (54.2— 66.8)	304	66.6 (60.0 —72.6)
**Socioeconomic factor**						
Household wealth index						
Rich	785	34.2 (29.6—38.9)	1276	26.6 (22.5 —31.1)	528	26.8 (21.5 —32.8)
Middle	3519	46.9 (44.7—49.2)	5,176	38.6 (36.4 —40.9)	2380	40.0 (37.5 —42.6)
Poor	6568	52.5 (50.7—54.2)	9,699	52.4 (50.4— 54.3)	3750	55.7 (53.6 —57.9)
**Mother's education**						
Secondary or higher	1554	37.6 (34.8—40.4)	2,813	30.0 (27.5 —32.6)	1590	28.8 (26.0 —31.8)
Primary	2111	48.4 (45.9—51.0)	2,637	42.0 (39.1 —45.0)	930	47.3 (43.6 —51.0)
No education	7207	52.2 (50.5—53.8)	10703	51.1 (49.2— 52.9)	4138	55.3 (53.2 —57.3)
**Mother's working status**						
Not working	4535	51.3 (49.4—53.3)	5778	47.3 (45.1— 49.4)	2592	50.2 (47.4 —52.9)
Working	6084	47.6 (45.9—49.4)	9535	44.7 (42.7 —46.7)	3901	46.1 (43.9 —48.3)
**Father's education**						
Secondary or higher	3022	44.2 (41.9—46.4)	5089	34.9 (32.9— 37.0)	2552	37.8 (35.2 —40.5)
Primary	1982	51.2 (48.2—54.2)	2520	49.0 (45.9 —52.2)	803	53.2 (48.6 —57.7)
No education	5589	51.6 (49.8—53.4)	8303	51.9 (50.0— 53.8)	2993	55.4 (53.0 —57.7)
**Number of women in household**						
One woman	6140	49.2 (47.5—50.8)	8972	43.9 (42.1 —45.7)	3702	44.2 (41.9 —46.4)
At least 2 women	4728	49.6 (47.8—51.5)	7181	48.5 (46.4 —50.5)	2956	52.4 (49.9 —54.9)
**Individual level factor (maternal)**						
Mother's age (years)						
< 20	686	46.3 (42.1— 50.4)	899	48.2 (44.3 —52.2)	336	49.4 (42.8— 56.0)
20 - 29	5347	50.5 (48.7— 52.2)	7951	45.7 (43.8 —47.6)	3233	46.5 (44.0— 49.0)

30 - 39	3609	48.7 (46.8— 50.6)	5654	45.1 (43.0— 47.1)	2453	47.9 (45.4 —50.5)
40 - 49	1231	48.3 (45.2 —51.4)	1648	48.6 (45.3 —51.9)	636	53.5 (48.6 —58.3)
**Mother's body mass index (kg/m2) (MBMI)**						
Underweight (MBMI < 18.5)	1585	58.5 (55.9 —61.1)	1589	53.3 (50.1 —56.5)	851	54.3 (50.0 —58.5)
Normal (18.5 ≤ MBMI ≤ 24.9)	7437	48.6 (46.9 —50.2)	11360	47.0 (45.3— 48.8)	4458	50.0 (47.5— 51.4)
Overweight or Obese (25 ≤ MBMI ≤ 29.9)/(MBMI ≥ 30)	1631	44.3 (41.4 —47.3)	3062	37.9 (35.6 —40.2)	1241	36.4 (32.9— 40.0)
**Birth order/ birth interval**						
First	1744	48.7 (46.1— 51.3)	2684	45.2 (42.6 —47.8)	1065	44.0 (40.6 —47.5)
2nd or 3rd rank, interval ≤ 2 yrs	863	51.2 (47.8— 54.6)	1244	48.0 (44.7— 51.4)	520	49.5 (44.6 —54.5)
2nd or 3rd rank, interval > 2 yrs	2432	48.3 (46.1— 50.4)	3643	43.1 (40.9 —45.5)	1426	42.9 (39.8 —46.1)
4th or higher rank, interval > 2 yrs	4420	48.3 (46.5 —50.2)	6484	45.5 (43.6— 47.4)	2756	48.0 (45.5— 50.4)
4th or higher rank, interval ≤ 2 yrs	1409	54.2 (50.8 —57.6)	2081	51.7 (48.5 —54.9)	882	59.1 (55.0 —63.1)
**Contraceptive use**						
Yes	726	40.7 (36.3 —45.2)	1052	35.3 (31.1 —39.7)	752	35.5 (31.4 —39.9)
No	10146	49.9 (48.5 —51.4)	15100	46.7 (45.0 —48.4)	5906	49.4 (47.5— 51.2)
**Maternal height (centimeter (CM))**						
≥ 160	3577	44.7 (42.8 —46.6)	5721	40.0 (38.0 —41.9)	2338	38.5 (35.9 —41.2)
155-159	3455	49.3 (47.2— 51.4)	5343	46.9 (44.6 —49.2)	2264	50.1 (47.3— 52.8)
150-154	2376	53.1 (50.8 —55.3)	3718	50.1 (47.5— 52.6)	1433	53.5 (50.4 —56.6)
145-149	997	55.1 (51.8 —58.4)	1040	55.7 (52.2— 59.2)	486	59.7 (54.9 —64.3)
< 145	344	54.9 (49.3— 60.2)	244	61.5 (53.1 —69.2)	77	73.2 (60.4— 83.1)
**Sex of child**						
Female	5548	46.6 (44.9— 48.3)	8179	44.0 (42.2— 45.8)	3292	44.6 (42.5 —46.8)
Male	5324	52.2 (50.5 — 53.9)	7973	47.9 (46.0 —49.8)	3366	50.9 (48.6 —53.3)
**Mother's perceived baby size**						
Average or larger	8982	48.3 (46.8 — 49.8)	13403	44.7 (43.0— 46.4)	5700	47.1 (45.2— 49.0)
Small or very small	1726	55.4 (52.5 — 58.2)	2498	52.0 (49.1 —54.9)	918	52.7 (48.6 —56.6)

Child's age (months)						
0 to 5	1118	22.9 (20.3— 25.7)	1581	18.7 (16.3 —21.4)	672	21.3 (17.8— 25.2)
6 to 11	1264	35.4 (32.1 —38.7)	1851	34.4 (31.5— 37.3)	750	28.9 (24.9— 33.1)
12 to 17	1269	55.9 (53.1— 58.8)	1967	45.0 (42.1 —48.0)	791	46.2 (41.6— 50.9)
18 to 23	825	62.5 (58.9 —65.9)	1271	52.1 (48.5 —55.6)	586	54.9 (50.3 —59.4)
24 to 29	1275	60.8 (57.6 —63.8)	1657	53.9 (50.4 —57.3)	706	60.2 (56.2— 64.0)
30 to 35	716	52.5 (48.2— 56.7)	1311	60.9 (57.4— 64.3)	569	62.4 (57.6 —67.0)
36 to 41	1477	51.7 (48.5 —54.9)	1935	51.8 (48.5— 55.1)	736	55.2 (51.1 —59.3)
42 to 47	803	51.7 (47.8 —55.5)	1356	53.6 (49.9— 57.3)	562	59.7 (54.5— 64.8)
48 to 53	1365	50.6 (47.6 —53.6)	1769	46.9 (43.3 —50.4)	712	45.5 (41.3— 49.8)
54 to 59	761	49.3 (47.9— 50.8)	1454	47.3 (43.4 —51.1)	572	50.8 (45.9— 55.7)
Health knowledge through (media exposure)						
**Frequency of listening to radio**						
At least once a week	2267	47.2 (44.2 —50.1)	4868	41.2 (38.9 —43.4)	1568	44.2 (40.7 —47.8)
Less than once a week	1475	49.5 (46.2 —52.7)	3733	47.2 (44.5— 50.0)	1418	43.5 (40.3— 46.8)
Never	4572	52.4 (48.9 —52.0)	7485	48.3 (46.3 —50.4)	3672	51.0 (48.8 —53.2)
**Frequency of reading newspaper or magazine**						
At least once a week	229	35.2 (28.7— 42.4)	571	25.7 (21.6 —30.2)	101	22.1 (14.2— 32.8)
Less than once a week	302	40.5 (34.2 —47.1)	691	31.9 (27.5 —36.7)	301	29.7 (22.4 —38.3)
Never	10116	50.1 (48.6 —51.5)	14771	47.3 (45.6— 49.0)	6256	49.1 (47.3— 50.9)
**Frequency of watching television**						
At least once a week	739	43.9 (39.6 —48.2)	3017	30.9 (28.6— 33.4)	1159	30.8 (27.1 —34.8)
Less than once a week	803	48.3 (43.6— 53.1)	2142	42.2 (38.7 —45.7)	884	37.9 (34.0 —42.0)
Never	8052	51.8 (50.2 —53.3)	10908	50.9 (49.0 —52.7)	4615	54.0 (52.0 —56.0)
Influence over household decision making						
**Woman has earning autonomy**						
By husband/partner alone or someone else	8640	49.7 (48.1— 51.3)	13182	47.2 (45.5— 48.9)	5626	49.5 (47.6— 51.3)
woman alone or joint decision	2232	47.9 (45.3— 50.6)	2970	40.4 (37.4— 43.4)	1031	39.0 (35.0— 43.1)

**Woman has healthcare autonomy**						
By husband/partner alone or someone else	7942	50.3 (48.6 —51.9)	12597	47.0 (45.2— 48.8)	5035	50.3 (48.4— 52.3)
woman alone or joint decision	2930	46.9 (44.7 —49.1)	3555	42.2 (39.6 —44.9)	1623	40.0 (37.0— 43.1)
**Woman has movement autonomy**						
By husband/partner alone or someone else	6014	49.6 (47.9 —51.8)	10913	47.3 (45.3 —49.3)	3456	51.1 (48.9 —53.4)
woman alone or joint decision	4858	48.7 (46.9 —50.6)	5240	43.0 (40.8 —45.2)	3202	44.3 (41.8— 46.8)
Health service related factor						
**Place of birth**						
Health facility	2038	39.4 (36.7— 42.1)	3528	33.4 (31.2 —35.7)	1833	33.8 (30.9 —36.9)
Home	8778	51.7 (50.2 —53.3)	12520	49.4 (47.6 —51.3)	4825	53.2 (51.2 —55.1)
**Mode of delivery**						
Non-caesarean	10780	49.4 (48.0— 50.9)	15900	46.1 (44.4 —47.7)	6568	48.2 (46.3 —50.0)
Caesarean	86	40.2 (29.4 —52.1)	157	31.7 (23.9 —40.6)	79	23.0 (14.5— 34.6)
**Delivery assistance**						
Health professional	2200	39.6 (37.1— 42.2)	3625	33.0 (30.7 —35.4)	1949	33.6 (30.8 —36.5)
Non-health professional	8564	51.9 (50.3— 53.5)	7074	53.6 (51.4 —55.8)	4486	54.3 (52.2 —56.3)
Immediate related factor						
**Dietary diversity score**						
< 5 foods/inadequate	9388	49.1 (47.6— 50.6)	14923	46.1 (44.4— 47.8)	6002	47.9 (46.0 —49.7)
≥ 5 foods/adequate	1484	50.9 (48.0— 53.8)	1229	43.3 (39.8— 46.8)	656	47.6 (43.1— 52.2)
**Initiation of breastfeeding**						
More than 1 hour after birth	9302	50.6 (49.1 —52.2)	13999	48.1 (46.4— 49.9)	5585	50.7 (48.8 —52.5)
Within 1 hour of birth	1570	41.7 (38.9— 44.6)	2153	31.6 (28.9 —34.3)	1073	33.0 (29.5 —36.6)
**Currently breastfeeding**						
No	580	51.7 (47.2 —56.1)	971	38.6 (35.1 —42.1)	384	37.8 (32.5 —43.4)
Yes	3895	41.8 (40.0 —43.7)	5700	36.9 (35.0 —38.9)	2416	37.3 (34.7 —40.0)
**Duration of breastfeeding**						
up to 12 months	6490	53.7 (52.0 —55.5)	10570	50.8 (48.7 —52.8)	4290	53.9 (51.8 —56.1)
more than 12 months	4382	42.9 (41.1 —44.7)	5583	36.7 (34.8 —38.7)	2368	36.8 (34.2 —39.5)

**Full vaccination**						
No	9805	49.7 (48.2— 51.2)	14168	47.1 (45.3— 48.8)	6004	48.7 (46.8 —50.6)
Yes	1067	46.1 (42.6 —49.7)	1984	37.8 (34.6— 41.3)	654	39.7 (35.4 —44.1)
Had diarrhea in the last 2 weeks						
No	9290	48.0 (46.5 —49.5)	14141	45.3 (43.7 —47.0)	5488	46.1 (44.3 —48.0)
Yes	1495	57.8 (54.6 —60.9)	1946	50.2 (47.2 —53.1)	1169	55.6 (52.1 —59.0)
Had fever in the last 2 weeks						
No	8957	48.6 (47.1 —50.2)	14075	45.4 (43.7 —47.1)	4675	45.8 (43.8 —47.9)
Yes	1819	53.4 (50.6 —56.1)	1987	50.1 (47.4 —52.9)	1981	52.5 (43.8 —47.9)

N, a weighted total of children who had valid date of birth and anthropometric measurement (height/length in centimeter) by each year of survey; NGZs, northern geopolitical zones; NDHS, Nigeria Demographic and Health Survey; PR, prevalence; CI, confidence interval; kg/m^2, kilogram/square meter; yrs, years.
