# Supplementary material for: Trends of Stunting Prevalence and Its Associated Factors among Nigerian Children Aged 0–59 Months Residing in the Northern Nigeria, 2008–2018
Source: Nutrients. 2021 Nov 29;13(12):4312. doi: 10.3390/nu13124312 (PMC8708583; doi:10.3390/nu13124312)
Supplement: Supplementary file 1 [file nutrients-13-04312-s001.zip › SupplementaryTable S2-nutients.pdf]

Table S2. Adjusted ORs (95% CI) for factors related to stunting in children aged 0–23 months of age in the NGZs, Nigeria

Variable	Model 1	Model 2	Model 3	Model 4	Model 5	Model 6	Model 7
**Community level factor**							
**Residence type**							
Urban	Ref	Ref		—	—	—	—
Rural	1.31 (1.02—1.69)^	0.95 (0.71—1.26)	—	—	—	—	—
**Geopolitical zones (North)**							
North Central	Ref	Ref	Ref	Ref	Ref	Ref	Ref
North East	2.05 (1.54—2.72)^	1.78 (1.32—2.39)^	1.85 (1.37—2.49)^	1.95 (1.43—2.66)^	2.02 (1.50—2.73)^	1.94 (1.44—2.62)^	1.91 (1.37—2.64)^
North West	2.56 (1.97—3.33)^	2.15 (1.62—2.84)^	2.38 (1.81—3.12)^	2.35 (1.78—3.11)^	2.48 (1.88—3.27)^	2.29 (1.73—3.03)^	2.36 (1.79—3.10)^
**Socioeconomic factor**							
Household wealth index							
Rich		Ref	Ref	Ref	Ref	Ref	Ref
Middle	—	1.56 (1.18—2.04)^	1.54 (1.19—1.99)^	1.52 (1.14—2.04)^	1.58 (1.21—2.05)^	1.49 (1.14—1.96)^	1.67 (1.27—2.21)^
Poor	—	1.76 (1.29—2.41)^	1.75 (1.34—2.29)^	1.76 (1.28—2.41)^	1.82 (1.38—2.40)^	1.67 (1.25—2.25)^	1.87 (1.39—2.51)^
**Mother's education**							
Secondary or higher		Ref				—	—
Primary	—	1.11 (0.83—1.47)	—	—	—	—	—
No education	—	1.42 (1.08—1.85)^	—	—	—	—	—
**Mother's working status**						—	—
Not working		Ref				—	—
Working	—	0.99 (0.83—1.20)	—	—	—	—	—
**Father's education**						—	—
Secondary or higher		Ref				—	—
Primary	—	1.18 (0.82—1.70)	—	—	—	—	—
No education	—	0.97 (0.75—1.26)	—	—	—	—	—
**Number of women in household**						—	—
One woman		Ref				—	—
At least 2 women	—	1.12 (0.94—1.34)	—	—	—	—	—
**Individual level factor (maternal)**						—	—
Mother's age (years)						—	—

< 20	—	—	0.78 (0.52—1.17)	—	—	—	—	
20 - 29	—	—	0.82 (0.64—1.04)	—	—	—	—	
30 - 39	—	—	Ref	—	—	—	—	
40 - 49	—	—	1.38 (0.98—1.95)	—	—	—	—	
**Mother's body mass index (kg/m2) (MBMI)**						—	—	
Underweight (MBMI < 18.5)	—		Ref	—	—	—	—	
Normal (18.5 ≤ MBMI ≤ 24.9)	—	—	0.85 (0.63—1.03)	—	—	—	—	
Overweight or Obese (25 ≤ MBMI ≤ 29.9)/(MBMI ≥ 30)	—	—	0.58 (0.42—1.03)	—	—	—	—	
**Birth order/ birth interval**						—	—	
First	—	—	1.27 (0.96—1.67)	—	—	—	—	
2nd or 3rd rank, interval ≤ 2 yrs	—	—	1.29 (0.87—1.91)	—	—	—	—	
2nd or 3rd rank, interval > 2 yrs	—		Ref	—	—	—	—	
4th or higher rank, interval > 2 yrs	—	—	1.09 (0.83—1.42)	—	—	—	—	
4th or higher rank, interval ≤ 2 yrs	—	—	1.38 (0.96—1.98)	—	—	—	—	
**Contraceptive use**						—	—	
Yes		—	Ref	—	—	—	—	
No		—	0.94 (0.72—1.23)	—	—	—	—	
**Maternal height (centimeter (CM))**								
≥ 160			Ref	Ref	Ref	Ref	Ref	
155-159	—	—	1.35 (1.06—1.72)^	1.30 (1.02—1.66)^	1.32 (1.04—1.68)^	1.32 (1.04—1.67)^	1.35 (1.06—1.72)^	
150-154	—	—	1.54 (1.18—1.99)^	1.48 (1.15—1.90)^	1.50 (1.17—1.92)^	1.50 (1.17—1.92)^	1.53 (1.20—1.96)^	
145-149	—	—	1.95 (1.38—2.75)^	1.81 (1.28—2.57)^	1.90 (1.35—2.69)^	1.92 (1.36—2.70)^	1.97 (1.38—2.80)^	
< 145	—	—	3.60 (1.78—7.29)^	3.57 (1.70—7.48)^	3.57 (1.77—7.22)^	3.67 (1.84—7.33)^	3.42 (1.70—6.87)^	
**Individual related factor** (Child)								
Sex **of child**								
Female	—	—	Ref	Ref	Ref	Ref	Ref	
**Male**	—	—	1.61 (1.37—1.91)^	1.65 (1.39—1.95)^	1.63 (1.38—1.92)^	1.63 (1.38—1.93)^	1.68 (1.43—1.99)^	
Mother's perceived baby **size**								
Average or larger	—	—	Ref	Ref	Ref	Ref	Ref	
Small or very small		—	—	1.43 (1.12—1.83)^	1.42 (1.11—1.83)^	1.45 (1.13—1.86)^	1.44 (1.13—1.84)^	1.50 (1.18—1.91)^

**Health knowledge through (media exposure**) Frequency of listening to radio								
At least once a week	—	—	—	Ref	—	—	—	
Less than once a week	—	—	—	0.80 (0.62—1.04)	—	—	—	
**Never**		—	—	—	0.85 (0.66—1.09)	—	—	—
Frequency of reading newspaper or **magazine**								
At least once a week	—	—	—	Ref	—	—	—	
Less than once a week	—	—	—	1.03 (0.42—2.51)	—	—	—	
**Never**	—	—	—	1.10 (0.50—2.39)	—	—	—	
Frequency of watching television								
At least once a week	—	—	—	Ref	—	—	—	
Less than once a week	—	—	—	1.08 (0.75—1.56)	—	—	—	
Never		—	—	—	1.30 (0.93—1.81)	—	—	—
**Influence over household decision** making		—	—					
Woman has earning autonomy			—	—				
By husband/partner alone or someone else	—	—	—	—	Ref	—	—	
**woman alone or joint** decision	—	—			1.02 (0.76—1.36)	—	—	
Woman has healthcare autonomy			—	—		—	—	
By husband/partner alone or someone else	—	—	—	—	Ref	—	—	
**woman alone or joint** decision	—	—	—	—	0.85 (0.66—1.11)	—	—	
Woman has movement autonomy						—	—	
By husband/partner alone or someone else	—	—	—	—	Ref	—	—	
woman alone or joint decision	—	—	—	—	1.17 (0.94—1.47)	—	—	
**Health service related** factor								
Place of **birth**								
Health facility	—	—	—	—	—	Ref	—	
**Home**	—	—	—	—	—	0.90 (0.58—1.40)	—	
Mode **of delivery**	—	—	—	—	—		—	

Non-caesarean				—	—	Ref	
**Caesarean**	—	—	—	—	—	0.99 (0.44—2.24)	—
Delivery assistance	—	—	—	—	—		—
Health professional	—	—	—	—	—	Ref	—
Non-health professional				—	—	1.38 (0.89—2.14)	
**Immediate related factor**	—	—	—				—
Dietary diversity score							—
< 5 foods/inadequate	—	—	—	—	—	—	Ref
≥ **5 foods**/adequate	—	—	—	—	—	—	1.92 (1.55—2.37)^
Initiation of breastfeeding	—	—	—	—	—	—	
More than 1 hour after birth							Ref
**Within 1** hour of birth	—	—	—	—	—	—	0.94 (0.78—1.13)
Currently **breastfeeding**	—	—	—	—	—	—	—
No	—	—	—	—	—	—	Ref
**Yes**							1.55 (0.84—2.86)
Duration of breastfeeding	—	—	—	—	—	—	—
up to 12 months	—	—	—	—	—	—	Ref
**more than 12** months	—	—	—	—	—	—	0.47 (0.26—0.83)
Full **vaccination**							
No	—	—	—	—	—	—	Ref
Yes	—	—	—	—	—	—	1.30 (1.03—1.64)^
Had diarrhea in the last 2 weeks	—	—	—	—	—	—	
No							Ref
Yes	—	—	—	—	—	—	1.08 (0.88—1.34)
Had fever in the last 2 weeks	—	—	—	—	—	—	
No	—	—	—	—	—	—	Ref
Yes	—	—	—	—	—	—	1.38 (0.89—2.14)

Notes: ^, significant variable (s) added to the next model; Model 1- Community level factors (residence type & region); Model 2 - significant variable(s) in Model 1 plus socioeconomic variables (household wealth status, maternal education, maternal work status, paternal education, number of women in the household); Model 3 – significant variable(s) in Model 2 plus individual level factor (mother's age, MBMI, contraceptive use, maternal height, perceived baby size by their mothers, child sex, & birth order/interval); Model 4 – significant variables in Model 3 plus health knowledge via media exposure (listening to radio, reading newspaper or magazine and watching television); Model 5 – significant variables in Model 4 plus household influence in decision making (power over earning, autonomy over healthcare and purchasing decision); Model 6 – significant variables in Model 5 plus health service related factor (delivery assistant, mode of delivery & place of delivery); Model 7 – significant variables in 6 plus immediate or direct factor (dietary diversity, early initiation of breastfeeding, currently breastfeeding, duration

of breastfeeding, vaccination, diarrhea in the last two weeks and fever in the last two weeks), yrs, years; OR (95%CI): Odds ratio with corresponding 95% confidence interval; Ref, reference category; NGZs, three northern geopolitical zones in Nigeria (northcentral, northeast and northwest).
