# Supplementary material for: Trends of Stunting Prevalence and Its Associated Factors among Nigerian Children Aged 0–59 Months Residing in the Northern Nigeria, 2008–2018
Source: Nutrients. 2021 Nov 29;13(12):4312. doi: 10.3390/nu13124312 (PMC8708583; doi:10.3390/nu13124312)
Supplement: Supplementary file 1 [file nutrients-13-04312-s001.zip › SupplementaryTable S3- nutrients.pdf]

Table S3. Adjusted ORs (95% CI) for factors related to stunting in children aged 24—59 months of age in the NGZs, Nigeria

**Variable**	**Model 1**	**Model 2**	**Model 3**	**Model 4**	**Model 5**	Model 6	**Model 7**
**Community level factor**							
**Residence type**							
Urban	Ref	Ref	Ref	Ref	Ref	Ref	Ref
Rural	2.03 (1.67—2.46)^	1.35 (1.10—1.66)^	1.38 (1.11—1.70)^	1.30 (1.05—1.61)^	1.29 (1.05—1.60)^	1.32 (1.07—1.63)^	1.37 (1.11—1.70)^
**Geopolitical zones (North)**							
North Central	Ref	Ref	Ref	Ref	Ref	Ref	Ref
North East	2.47 (1.96—3.11)^	1.98 (1.58—2.48)^	2.07 (1.62—2.64)^	1.98 (1.56—2.50)^	1.92 (1.53—2.42)^	2.01 (1.59—2.55)^	1.96 (1.55—2.48)^
North West	3.99 (3.21—4.96)^	3.20 (2.57—3.99)^	3.26 (2.59—4.11)^	3.06 (2.45—3.83)^	3.03 (2.42—3.78)^	3.10 (2.47—3.88)^	3.23 (2.58—4.04)^
**Socioeconomic factor**							
Household wealth index							
Rich		Ref	Ref	Ref	Ref	Ref	Ref
Middle	—	1.59 (1.26—2.01)^	1.53 (1.21—1.93)^	1.46 (1.16—1.85)^	1.49 (1.19—1.88)^	1.53 (1.22—1.93)^	1.55 (1.24—1.95)^
Poor	—	1.54 (1.19—2.00)^	1.47 (1.14—1.90)^	1.39 (1.08—1.80)^	1.42 (1.10—1.83)^	1.48 (1.15—1.91)^	1.49 (1.15—1.91)^
**Mother's education**							
Secondary or higher		Ref	Ref	Ref	Ref	Ref	Ref
Primary	—	1.74 (1.33—2.27)^	1.84 (1.38—2.46)^	1.71 (1.27—2.30)^	1.63 (1.21—2.21)^	1.68 (1.25—2.25)^	1.76 (1.31—2.35)^
No education	—	1.85 (1.44—2.38)^	1.94 (1.52—2.46)^	1.73 (1.34—2.22)^	1.63 (1.27—2.10)^	1.71 (1.32—2.22)^	1.90 (1.48—2.44)^
**Mother's working status**						—	—
Not working		Ref				—	—
Working	—	0.94 (0.80—1.10)	—	—	—	—	—
**Father's education**						—	—
Secondary or higher		Ref				—	—
Primary	—	1.14 (0.88—1.48)	—	—	—	—	—
No education	—	1.16 (0.93—1.44)	—	—	—	—	—
**Number of women in household**						—	—
One woman		Ref				—	—

At least 2 women	—	1.08 (0.91—1.28)	—	—	—	—	—
**Individual level factor (maternal)**						—	—
Mother's age (years)						—	—
< 20	—	—	1.24 (0.65—2.35)	—	—	—	—
20 - 29	—	—	0.98 (0.78—1.22)	—	—	—	—
30 - 39	—	—	Ref	—	—	—	—
40 - 49	—	—	0.99 (0.75—1.31)	—	—	—	—
**Mother's body mass index (kg/m2) (MBMI)**						—	—
Underweight (MBMI < 18.5)	—		Ref	—	—	—	—
Normal (18.5 ≤ MBMI ≤ 24.9)	—	—	0.91 (0.68—1.21)	—	—	—	—
Overweight or Obese (25 ≤ MBMI ≤ 29.9)/(MBMI ≥ 30)	—	—	0.71 (0.51—1.00)	—	—	—	—
**Birth order/ birth interval**						—	—
First	—	—	0.99 (0.77—1.26)	0.99 (0.78—1.25)	0.99 (0.78—1.25)	1.03 (0.81—1.30)	1.01 (0.80—1.28)
2nd or 3rd rank, interval ≤ 2 yrs	—	—	1.35 (0.99—1.84)	1.36 (1.00—1.86)	1.38 (1.01—1.88)	1.34 (0.99—1.81)	1.32 (0.98—1.79)
2nd or 3rd rank, interval > 2 yrs	—		Ref	Ref	Ref	Ref	Ref
4th or higher rank, interval > 2 yrs	—	—	1.06 (0.83—1.37)	1.04 (0.84—1.30)	1.06 (0.85—1.31)	1.03 (0.83—1.27)	1.01 (0.81—1.26)
4th or higher rank, interval ≤ 2 yrs	—	—	1.52 (1.15—2.02)^	1.53 (1.19—1.98)^	1.52 (1.18—1.96)^	1.48 (1.15—1.91)^	1.46 (1.13—1.88)^
**Contraceptive use**						—	—
Yes		—	Ref	—	—	—	—
No		—	1.16 (0.90—1.49)	—	—	—	—
**Maternal height (centimeter (CM))**							
≥ 160			Ref	Ref	Ref	Ref	Ref
155-159	—	—	1.94 (1.61—2.35)^	1.93 (1.59—2.33)^	1.96 (1.62—2.37)^	1.94 (1.61—2.34)^	1.93 (1.60—2.32)^
150-154	—	—	2.00 (1.61—2.48)^	2.03 (1.63—2.53)^	2.04 (1.64—2.54)^	2.01 (1.62—2.50)^	2.01 (1.62—2.49)^
145-149	—	—	2.47 (1.78—3.42)^	2.42 (1.76—3.40)^	2.37 (1.73—3.26)^	2.40 (1.74—3.30)^	2.39 (1.74—3.29)^
< 145	—	—	3.57 (1.45—8.83)^	4.46 (1.88—10.6)^	4.61 (1.99—10.7)^	3.12 (1.33—7.33)^	2.86 (1.24—6.59)^
Individual related factor (Child)							
**Sex of child**							

Female	—	—	Ref	Ref	Ref	Ref	Ref
Male	—	—	1.22 (1.04—1.43)^	1.22 (1.04—1.43)^	1.20 (1.02—1.41)^	1.23 (1.05—1.44)^	1.22 (1.04—1.43)^
**Mother's perceived baby size**							
Average or larger	—	—	Ref	—	—	—	—
Small or very small	—	—	1.00 (0.79—1.26)	—	—	—	—
Health knowledge through (media exposure)							
**Frequency of listening to radio**							
At least once a week	—	—	—	Ref	—	—	—
Less than once a week	—	—	—	0.86 (0.68—1.11)	—	—	—
Never	—	—	—	0.86 (0.69—1.08)	—	—	—
**Frequency of reading newspaper or magazine**							
At least once a week	—	—	—	Ref	Ref	Ref	Ref
Less than once a week	—	—	—	2.00 (0.93—4.30)	2.04 (0.95—4.35)	2.09 (1.00—4.37)	2.02 (0.98—4.14)
Never	—	—	—	2.15 (1.11—4.17)^	2.07 (1.07—4.00)^	2.03 (1.08—3.84)^	2.07 (1.11—3.84)^
**Frequency of watching television**							
At least once a week	—	—	—	Ref	Ref	—	—
Less than once a week	—	—	—	1.07 (0.78—1.48)	1.01 (0.74—1.37)	—	—
Never	—	—	—	1.43 (1.08—1.90)^	1.30 (1.00—1.69)	—	—
Influence over household decision making			—	—			
**Woman has earning autonomy**			—	—			
By husband/partner alone or someone else	—	—	—	—	Ref	—	—
woman alone or joint decision	—	—			0.91 (0.72—1.15)	—	—
**Woman has healthcare autonomy**			—	—		—	—
By husband/partner alone or someone else	—	—	—	—	Ref	—	—
woman alone or joint decision	—	—	—	—	0.88 (0.71—1.10)	—	—
**Woman has movement autonomy**						—	—
By husband/partner alone or someone else	—	—	—	—	Ref	—	—
woman alone or joint decision	—	—	—	—	0.91 (0.75—1.11)	—	—

Health service related factor							
**Place of birth**							
Health facility	—	—	—	—	—	Ref	—
Home	—	—	—	—	—	1.00 (0.70—1.42)	—
**Mode of delivery**	—	—	—	—	—		—
Non-caesarean				—	—	Ref	
Caesarean	—	—	—	—	—	0.58 (0.29—1.19)	—
**Delivery assistance**	—	—	—	—	—		—
Health professional	—	—	—	—	—	Ref	—
Non-health professional				—	—	1.25 (0.81—1.82)	
Immediate related factor	—	—	—				—
**Full vaccination**							
No	—	—	—	—	—	—	Ref
Yes	—	—	—	—	—	—	1.28 (0.97—1.69)
Had diarrhea in the last 2 weeks	—	—	—	—	—	—	
No							Ref
Yes	—	—	—	—	—	—	1.69 (1.34—2.15)^
Had fever in the last 2 weeks	—	—	—	—	—	—	
No	—	—	—	—	—	—	Ref
Yes	—	—	—	—	—	—	0.98 (0.82—1.18)

Notes: ^, significant variable (s) added to the next model; Model 1- Community level factors (residence type & region); Model 2 - significant variable(s) in Model 1 plus socioeconomic variables (household wealth status, maternal education, maternal work status, paternal education, number of women in the household); Model 3 – significant variable(s) in Model 2 plus individual level factor (mother's age, MBMI, contraceptive use, maternal height, perceived baby size by their mothers, child sex, & birth order/interval); Model 4 – significant variables in Model 3 plus health knowledge via media exposure (listening to radio, reading newspaper or magazine and watching television); Model 5 – significant variables in Model 4 plus household influence in decision making (power over earning, autonomy over healthcare and purchasing decision); Model 6 – significant variables in Model 5 plus health service related factor (delivery assistant, mode of delivery & place of delivery); Model 7 – significant variables in 6 plus immediate or direct factor (vaccination, diarrhea in the last two weeks and fever in the last two weeks), yrs, years; OR (95%CI): Odds ratio with corresponding 95% confidence interval; Ref, reference category; NGZs, three northern geopolitical zones in Nigeria (northcentral, northeast and northwest).
