# Supplementary material for: Trends of Stunting Prevalence and Its Associated Factors among Nigerian Children Aged 0–59 Months Residing in the Northern Nigeria, 2008–2018
Source: Nutrients. 2021 Nov 29;13(12):4312. doi: 10.3390/nu13124312 (PMC8708583; doi:10.3390/nu13124312)
Supplement: Supplementary file 1 [file nutrients-13-04312-s001.zip › SupplementaryTable S4-nutrients.pdf]

Table S4. Adjusted ORs (95% CI) for factors related to stunting in children aged 0-59 months of age in the NGZs, Nigeria

Variable	Model 1	Model 2	Model 3	Model 4	Model 5	Model 6	Model 7
**Community level factor**							
**Residence type**							
Urban	Ref	Ref	—	—	—	—	—
Rural	1.65 (1.40—1.94)^	1.14 (0.97—1.34)	—	—	—	—	—
**Geopolitical zones (North)**							
North Central	Ref	Ref	Ref	Ref	Ref	Ref	Ref
North East	2.23 (1.84—2.71)^	1.85 (1.53—2.25)^	1.87 (1.53—2.27)^	1.83 (1.50—2.23)^	1.82 (1.49—2.21)^	1.79 (1.47—2.19)^	1.74 (1.40—2.15)^
North West	3.23 (2.68—3.88)^	2.63 (2.18—3.18)^	2.64 (2.19—3.18)^	2.50 (2.07—3.02)^	2.51 (2.08—3.04)^	2.45 (2.02—3.97)^	2.48 (2.04—3.03)^
**Socioeconomic factor**							
Household wealth index							
Rich		Ref	Ref	Ref	Ref	Ref	Ref
Middle	—	1.54 (1.29—1.84)^	1.57 (1.32—1.87)^	1.50 (1.24—1.80)^	1.50 (1.25—1.80)^	1.47 (1.22—1.77)^	1.52 (1.26—1.84)^
Poor	—	1.59 (1.31—1.94)^	1.65 (1.37—2.00)^	1.57 (1.29—1.92)^	1.57 (1.21—1.92)^	1.51 (1.23—1.85)^	1.52 (1.23—1.88)^
**Mother's education**							
Secondary or higher		Ref	Ref	Ref	Ref	Ref	Ref
Primary	—	1.49 (1.23—1.81)^	1.48 (1.21—1.81)^	1.47 (1.20—1.81)^	1.48 (1.21—1.81)^	1.45 (1.18—1.77)^	1.43 (1.15—1.77)^
No education	—	1.71 (1.44—2.04)^	1.69 (1.43—2.00)^	1.61 (1.35—1.92)^	1.62 (1.37—1.91)^	1.53 (1.28—1.82)^	1.54 (1.28—1.84)^
**Mother's working status**						—	—
Not working		Ref				—	—
Working	—	1.02 (0.90—1.15)	—	—	—	—	—
**Father's education**						—	—
Secondary or higher		Ref				—	—
Primary	—	1.14 (0.91—1.42)	—	—	—	—	—
No education	—	1.06 (0.90—1.25)	—	—	—	—	—
**Number of women in household**						—	—
One woman		Ref				—	—
At least 2 women	—	1.12 (0.98—1.27)	—	—	—	—	—
**Individual level factor (maternal)**						—	—
Mother's age (years)						—	—
< 20	—	—	0.70 (0.51—1.01)	—	—	—	—

20 - 29	—	—	0.98 (0.78—1.22)	—	—	—	—
30 - 39	—	—	Ref	—	—	—	—
40 - 49	—	—	1.17 (0.94—1.45)	—	—	—	—
**Mother's body mass index (kg/m2) (MBMI)**						—	—
Underweight (MBMI < 18.5)	—		Ref	—	—	—	—
Normal (18.5 ≤ MBMI ≤ 24.9)	—	—	0.91 (0.76—1.10)	—	—	—	—
Overweight or Obese (25 ≤ MBMI ≤ 29.9)/(MBMI ≥ 30)	—	—	0.72 (0.57—1.05)	—	—	—	—
**Birth order/ birth interval**						—	—
First	—	—	1.20 (1.00—1.43)	1.11 (0.93—1.31)	1.10 (0.93—1.31)	1.13 (0.95—1.34)	1.16 (0.97—1.38)
2nd or 3rd rank, interval ≤ 2 yrs	—	—	1.40 (1.10—1.78)^	1.38 (1.09—1.76)^	1.40 (1.10—1.78)^	1.39 (1.09—1.77)^	1.30 (1.02—1.66)^
2nd or 3rd rank, interval > 2 yrs	—		Ref	Ref	Ref	Ref	Ref
4th or higher rank, interval > 2 yrs	—	—	0.97 (0.80—1.17)	1.07 (0.91—1.25)	1.07 (0.92—1.25)	1.07 (0.92—1.25)	1.09 (0.94—1.29)
4th or higher rank, interval ≤ 2 yrs	—	—	1.53 (1.22—1.93)^	1.67 (1.35—2.06)^	1.66 (1.34—2.05)^	1.66 (1.34—2.05)^	1.53 (1.24—1.89)^
**Contraceptive use**						—	—
Yes		—	Ref	—	—	—	—
No		—	1.04 (0.86—1.27)	—	—	—	—
**Maternal height (centimeter (CM))**							
≥ 160			Ref	Ref	Ref	Ref	Ref
155-159	—	—	1.61 (1.40—1.86)^	1.57 (1.35—1.82)^	1.58 (1.37—1.83)^	1.58 (1.37—1.83)^	1.66 (1.43—1.93)^
150-154	—	—	1.70 (1.46—1.99)^	1.69 (1.45—1.97)^	1.69 (1.45—1.97)^	1.68 (1.44—1.96)^	1.78 (1.52—2.09)^
145-149	—	—	2.10 (1.67—2.64)^	2.05 (1.62—2.59)^	2.04 (1.62—2.57)^	2.04 (1.62—2.57)^	2.18 (1.72—2.77)^
< 145	—	—	3.32 (1.86—5.92)^	3.92 (2.24—6.87)^	3.98 (2.28—6.95)^	4.11 (2.36—7.17)^	4.05 (2.24—7.31)^
Individual related factor (Child)							
**Sex of child**							
Female	—	—	Ref	Ref	Ref	Ref	Ref
Male	—	—	1.34 (1.19—1.51)^	1.35 (1.20—1.53)^	1.33 (1.18—1.51)^	1.34 (1.19—1.52)^	1.37 (1.21—1.55)^
**Mother's perceived baby size**							
Average or larger	—	—	Ref	—	—	—	—
Small or very small	—	—	1.09 (0.91—1.31)	—	—	—	—
Health knowledge through (media exposure)							
**Frequency of listening to radio**							

At least once a week	—	—	—	Ref	—	—	—
Less than once a week	—	—	—	0.83 (0.69—1.01)	—	—	—
Never	—	—	—	0.85 (0.72—1.00)	—	—	—
**Frequency of reading newspaper or magazine**							
At least once a week	—	—	—	Ref	—	—	—
Less than once a week	—	—	—	1.46 (0.81—2.64)	—	—	—
Never	—	—	—	1.50 (0.90—2.52)^	—	—	—
**Frequency of watching television**							
At least once a week	—	—	—	Ref	Ref	Ref	Ref
Less than once a week	—	—	—	1.07 (0.84—1.38)	1.02 (0.80—1.29)	1.01 (0.80—1.28)	1.04 (0.81—1.32)
Never	—	—	—	1.37 (1.11—1.68)^	1.26 (1.04—1.52)^	1.23 (1.02—1.49)^	1.32 (1.08—1.61)^
Influence over household decision making			—	—			
**Woman has earning autonomy**			—	—			
By husband/partner alone or someone else	—	—	—	—	Ref	—	—
woman alone or joint decision	—	—			0.97 (0.80—1.18)	—	—
**Woman has healthcare autonomy**			—	—		—	—
By husband/partner alone or someone else	—	—	—	—	Ref	—	—
woman alone or joint decision	—	—	—	—	0.88 (0.74—1.05)	—	—
**Woman has movement autonomy**						—	—
By husband/partner alone or someone else	—	—	—	—	Ref	—	—
woman alone or joint decision	—	—	—	—	1.05 (0.90—1.22)	—	—
Health service related factor							
**Place of birth**							
Health facility	—	—	—	—	—	Ref	—
Home	—	—	—	—	—	0.91 (0.69—1.20)	—
**Mode of delivery**	—	—	—	—	—		—
Non-caesarean				—	—	Ref	
Caesarean	—	—	—	—	—	0.72 (0.40—1.31)	—
**Delivery assistance**	—	—	—	—	—		—
Health professional	—	—	—	—	—	Ref	Ref
Non-health professional				—	—	1.33 (1.00—1.78)	1.27 (1.06—1.51)^

Immediate related factor	—	—	—				—
**Dietary diversity score**							—
< 5 foods/inadequate	—	—	—	—	—	—	Ref
≥ 5 foods/adequate	—	—	—	—	—	—	1.85 (1.48—2.32)
**Initiation of breastfeeding**	—	—	—	—	—	—	
More than 1 hour after birth							Ref
Within 1 hour of birth	—	—	—	—	—	—	0.86 (0.72—1.04)
**Duration of breastfeeding**	—	—	—	—	—	—	—
up to 12 months	—	—	—	—	—	—	Ref
more than 12 months	—	—	—	—	—	—	0.40 (0.33—0.47)
**Full vaccination**							
No	—	—	—	—	—	—	Ref
Yes	—	—	—	—	—	—	1.07 (0.87—1.33)
Had diarrhea in the last 2 weeks	—	—	—	—	—	—	
No							Ref
Yes	—	—	—	—	—	—	1.47 (1.25—1.73)^
Had fever in the last 2 weeks	—	—	—	—	—	—	
No	—	—	—	—	—	—	Ref
Yes	—	—	—	—	—	—	1.03 (0.90—1.18)

Notes: ^, significant variable (s) added to the next model; Model 1- Community level factors (residence type & region); Model 2 - significant variable(s) in Model 1 plus socioeconomic variables (household wealth status, maternal education, maternal work status, paternal education, number of women in the household); Model 3 – significant variable(s) in Model 2 plus individual level factor (mother's age, MBMI, contraceptive use, maternal height, perceived baby size by their mothers, child sex, & birth order/interval); Model 4 – significant variables in Model 3 plus health knowledge via media exposure (listening to radio, reading newspaper or magazine and watching television); Model 5 – significant variables in Model 4 plus household influence in decision making (power over earning, autonomy over healthcare and purchasing decision); Model 6 – significant variables in Model 5 plus health service related factor (delivery assistant, mode of delivery & place of delivery); Model 7 – significant variables in 6 plus immediate or direct factor (dietary diversity, early initiation of breastfeeding, duration of breastfeeding, vaccination, diarrhea in the last two weeks and fever in the last two weeks), yrs, years; OR (95%CI): Odds ratio with corresponding 95% confidence interval; Ref, reference category; NGZs, three northern geopolitical zones in Nigeria (northcentral, northeast and northwest).
